# A Problem-Setup-Centric Framework for Mixed-Variable Chemical Formulation Optimization with Customized Constraints

**DOI:** 10.3390/ma19153248

**Published:** 2026-07-31

**Authors:** Yingjun Zhang, Shaochen Chen, Suqi Gao, Pengcheng Xu, Wencong Lu

**Affiliations:** 1 Department of Chemistry, Shanghai University, Shanghai 200444, China; zyjzhang@shu.edu.cn; 2 Hubei Institute of Aerospace Chemotechnology, Xiangyang 441003, China; chenscx@163.com (S.C.); gaosuqi@casc42.cn (S.G.)

**Keywords:** virtual formulation generation, multi-objective optimization, tolerance-aware design, surrogate modeling

## Abstract

Chemical formulation design is a challenging multi-objective optimization problem involving mixed continuous and discrete variables, feasibility constraints, and limited experimental data. Existing virtual formulation generation methods are often restricted to interpolation within historically observed regions and therefore lack sufficient exploratory capability. In this work, a problem-setup-centric multi-objective optimization framework for virtual chemical formulation generation is proposed. The framework uses a pymoo-based mixed-variable evolutionary workflow to optimize machine-learning-predicted properties under application-specific objectives and constraints. One energetic-material formulation dataset and one steel-alloy composition dataset are used to evaluate the proposed approach. The generated Pareto solutions include candidates that remain similar to historical formulations and candidates that show greater statistical deviation from the historical data distribution. The t-SNE projections provide qualitative visualizations of local structural relationships, while Mahalanobis-distance and feature-distribution analyses characterize statistical deviation in the original feature space. Overall, this study provides a flexible and practical workflow for data-driven virtual formulation design with customized mixed-variable constraints.

## 1. Introduction

Chemical formulation and materials design integrate structure–property relationships, process constraints, and functional requirements to rationally design manufacturable material systems for applications in energy storage, coatings, propellant, polymers, and pharmaceuticals [1,2,3,4,5]. In practical applications, this task is inherently highly challenging, as ideal products must simultaneously satisfy multiple competing objectives—including performance, safety, cost, stability, and manufacturability—under limited experimental data and complex feasibility constraints. The combinatorial explosion of composition spaces, together with the high cost, long cycle time, and operational risks of wet-lab experiments, has driven the widespread adoption of computational methods in formulation discovery [6,7].

An important development direction is virtual sample generation, which uses data-driven surrogate models or generative methods to propose candidate formulations in silico before physical validation [8,9,10]. Such approaches can substantially broaden search coverage while reducing experimental burden. However, many existing methods remain fundamentally conservative: they generate candidates primarily through interpolation within the support of historical observations or through local perturbations around known recipes [11,12,13,14,15]. As a result, the generated candidates often inherit the limitations of historical data and may fail to discover solutions with substantial performance improvements. In practical formulation engineering, valuable innovations are frequently located near—or moderately beyond—the boundary of historically explored design regions rather than strictly inside them [8,16,17]. Thus, the central challenge is not merely to generate more plausible samples, but also to identify candidates beyond densely represented historical regions while assessing their statistical deviation, prediction uncertainty, and engineering relevance [18,19,20].

Motivated by these challenges, multi-objective optimization (MOO) provides a natural framework for balancing competing criteria by identifying Pareto solution sets rather than reducing multiple objectives to a single scalar value [21]. Established strategies include scalarization-based methods [22,23], Pareto-based evolutionary algorithms such as NSGA and SPEA2 [24,25], and decomposition-based approaches such as MOEA/D [26,27]. Recent studies further combine ML/AI with chemical design through LLM-assisted evolutionary optimization for propellant formulations [28], staged diversity control for reaction-condition optimization [29], and generative inverse design of catalyst ligands [30]. These studies illustrate the growing range of data-driven strategies available for chemical-space exploration.

Applying these approaches to practical formulation design remains challenging when the available data are limited and the design space contains mixed variable types and multiple domain constraints. Continuous variable bounds and admissible discrete choices often need to be defined using historical data and domain knowledge, while compositional, operational, and safety requirements require explicit constraint formulations [31,32,33,34,35,36,37]. Without appropriate problem configuration, the resulting Pareto solutions may satisfy numerical bounds but remain poorly aligned with engineering requirements and historically validated formulation patterns [34,38,39,40,41].

To address this problem, the present study develops a problem-setup-centric framework for multi-objective formulation design in mixed continuous–discrete spaces. Rather than introducing a new optimization algorithm, the framework focuses on translating practical formulation requirements into an executable optimization problem by defining variable representations, predicted objectives, and numerical equality and inequality constraints. These components are implemented through an application-specific layer built on pymoo, while the evolutionary search uses existing mixed-variable optimization components. The framework is evaluated using an energetic-material formulation dataset and a steel-alloy composition dataset. The results show that it generates Pareto solutions under the specified variable ranges and constraints while distinguishing candidates with different degrees of statistical deviation from the historical data distribution.

## 2. Materials and Methods

**Figure 1:**
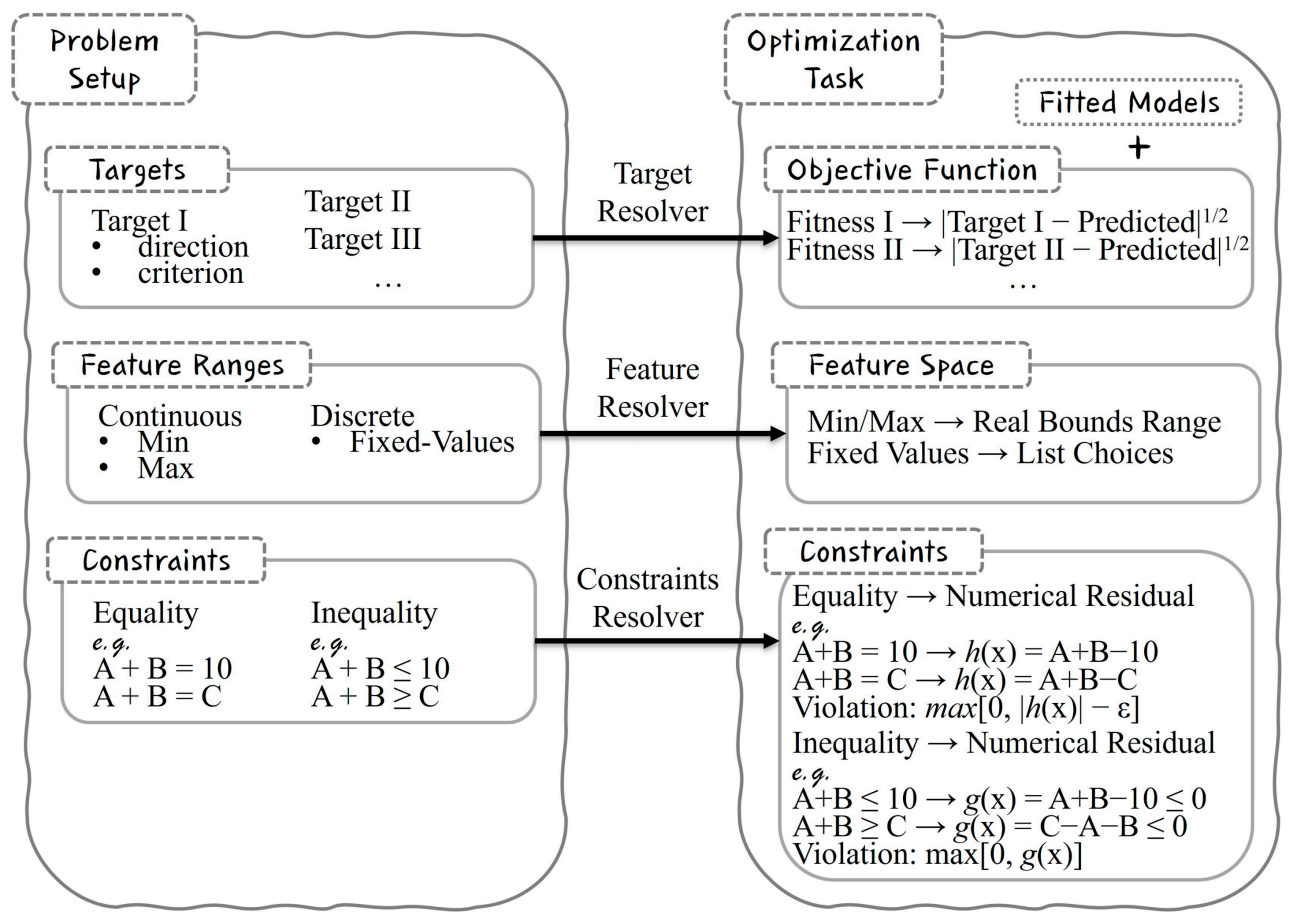
Problem setup and Optimization task.

This section outlines the overall methodology, which is summarized schematically in Figure 1. The mixed-variable optimization problem is formally defined in Section 2.1, and the corresponding optimization task—including the objective function and constraint handling—is detailed in Section 2.2. Section 2.3 introduces the two test cases with their experimental data, and Section 2.4 describes the algorithmic implementation using the pymoo framework, the archiving strategy, and the performance metrics adopted for evaluation.

### 2.1. Problem Setup

The candidate solutions in a practical formulation design task are usually characterized by a mixture of continuous and discrete variables. Such mixed-variable structures are common in chemical formulation problems, where continuous variables represent component proportions or process conditions, while discrete variables encode categorical choices such as material grades, processing modes, or manufacturing routes.

Formally, let $x\in R^{d_{c}}\times I^{d_{d}}$ denote a mixed-variable formulation vector with $d_{c}$ continuous and $d_{d}$ discrete variables. In principle, one formulation is often associated with many performance indicators, which are treated as targets in the formulation design task. The target vector $\hat{y}\left(x\right)$ is then defined as:

(1) $$\hat{y}\left(x\right)=\left({\hat{y}}_{1}\left(x\right),{\hat{y}}_{2}\left(x\right),\ldots \right)\in R$$

where each target $\hat{y}\left(x\right)$ corresponds to a key performance indicator selected for optimization.

Equality and inequality constraints are evaluated using numerical residuals. Each equality constraint is rearranged into the standard form $h\left(x\right)=0$. For example, A+B=10 is converted to $h\left(x\right)=A+B-10$, and the magnitude $max\;[0,\;\left|h\left(x\right)\right|-\epsilon ]$ ($\epsilon =10^{-4}$) represents the degree of equality-constraint violation. Each inequality constraint is expressed in the form $g\left(x\right)\leq 0$. Thus, A+B≤10 is converted to $g\left(x\right)=A+B-10$, whereas A+B≥C is converted to $g\left(x\right)=C-A-B$. An inequality constraint is feasible when $g\left(x\right)\leq 0$, and its violation is quantified as max [0, g(x)]. The resulting numerical residuals are supplied to the optimizer as the equality-constraint vector h(x) and the inequality-constraint vector g(x). In the pymoo implementation, these vectors correspond to out[“H”] and out[“G”] provided by pymoo’s Problem modules, respectively. This representation preserves the magnitude of each constraint violation and allows the constrained selection procedure to distinguish slightly infeasible candidates from severely infeasible candidates.

For the continuous feature $x_{j}$, lower and upper bounds $l_{x_{j}},u_{x_{j}}$ are estimated from historical data (e.g., quantile range after outlier trimming), while the set $S_{t}$ specifies admissible fixed values or optional constraints for the discrete feature $x_{t}$. Thus, the resulting feasible formulation set could be defined as:

(2) $$X_{feas}=\left\{x|l_{x_{j}}\leq x_{j}\leq u_{x_{j}},x_{t}\in S_{t},g\left(x\right)\leq 0,h_{i}\left(x\right)=0\right\}$$

### 2.2. Optimization Task

Given the abundance of existing third-party optimization libraries, the focus is on defining the optimization task, with the objective function as the core. The number of values returned by the (multi-)objective function equals the number of targets, where each returned value is defined as the square root of the absolute error between the predicted value from external machine learning models and the corresponding desired target value. Thus, minimizing this objective function drives the formulation predictions ever closer to the desired targets.

In the problem setup, continuous features are specified by minimum and maximum values that define their lower and upper bounds, whereas discrete features are represented as finite sets of selectable values. For constraint handling, equality and inequality constraints are converted into numerical residuals, as described in Section 2.1. Equality constraints are expressed in the form h(x), whereas inequality constraints are expressed as g(x). These residuals retain the magnitude of constraint violations and are supplied to the optimizer rather than being reduced to Boolean values.

### 2.3. Test Problem: Formulation Design Data

Case I: Multi-component energetic material formulation [42]. The first case comprises 137 formulation records, each characterized by the mass percentages of 14 ingredients, including NC + NG, burning rate inhibitor, catalyst, nAl, RDX, DEP, Al_2_O_3_, HMX, mAl, CL-20, nDPN, nNi, Al, and “others” (a variable). The total ingredient percentage (SUM) is maintained at approximately 100%, enforced as an equality constraint on the sum of these 14 ingredients. Additionally, the operating pressure (Pressure) is treated as a continuous formulation variable, as it directly influences the burning behavior and is subject to optimization. An inequality constraint, Pressure ≥ 10, is introduced to demonstrate the handling of inequality constraints within the framework.

Burning rate inhibitor, RDX, DEP, Al_2_O_3_, HMX, nDPN, Al, and CL-20 are treated as discrete features because they each take only two distinct values in the dataset. The remaining ingredients, along with Pressure, are modeled as continuous features.

The performance indicator Burning rate ($y_{1}$) is selected as the optimization target. To construct a bi-objective optimization task with a conflicting trend, a second indicator, Burning rate II ($y_{2}$), is artificially created from $y_{1}$ as:

(3) $$y_{2}=exp(max(y_{1})-y_{1}/10)$$

where $max(y_{1})$ is the maximum $y_{1}$. By this definition, an increase in $y_{1}$ leads to a decrease in $y_{2}$, thereby creating a pair of objectives that cannot be simultaneously optimized and introducing the necessary trade-off for multi-objective optimization. The second objective in Case I is introduced to provide a controlled illustration of the bi-objective optimization workflow. As it is deterministically derived from the first objective, this case is not interpreted as evidence that the framework captures an engineering trade-off between independent material properties.

Case II: Steel-alloy composition dataset [43]. The second case contains 587 steel-alloy records, each described by the weight percentages of 16 elements: Fe, Mn, Cr, Ni, Si, C, Mo, S, P, Cu, V, Nb, N, Al, Ti, and W. Elongation ($y_{1}$) and tensile strength $y_{2}$ are selected as two separately reported optimization targets. The two properties exhibit a negative Pearson correlation of approximately −0.613, reflecting a practical strength–ductility trade-off. Among 16 elements, P and Al are treated as discrete features, while the remaining 14 elements are continuous features. During optimization, the sum of the 16 elemental fractions is normalized to 100% through an equality constraint. Separate predictive models are trained to predict elongation and tensile strength, and both properties are maximized simultaneously.

### 2.4. Optimization Algorithm Implementation

The optimization tasks defined in Section 2.2 are implemented as an application-specific extension built on pymoo. Rather than directly applying pymoo’s off-the-shelf NSGA-II class, the implementation uses pymoo’s MixedVariableGA to accommodate the mixed continuous–discrete formulation space. Continuous variables are represented using pymoo’s Real variables with user-defined or historically derived bounds, whereas discrete variables are represented using Choice variables restricted to predefined admissible value sets. The mixed-variable mating, variation, non-dominated sorting, and rank-and-crowding survival mechanisms are provided by pymoo.

For each candidate x, the objective values are computed as:

(4) $$f_{i}\left(x\right)=\sqrt{\left|{\hat{y}}_{i}\left(x\right)-y_{i}^{des}\right|},\;i=1,\;2,\;\ldots$$

where ${\hat{y}}_{i}\left(x\right)$ is the machine-learning-predicted value and $y_{i}^{des}$ is the desired target.

Two types of search outputs are retained: the final Pareto solutions from each individual run and the intermediate Pareto candidates in their respective generations. The intermediate Pareto candidates may remain useful for practical formulation design even though some candidates are not retained in the final Pareto front. The available data, formulated objectives, and mathematical constraints may not fully capture all practical considerations, including manufacturability, material availability, safety preferences, and other domain-specific requirements that are difficult to quantify. These intermediate Pareto candidates therefore provide a broader candidate pool for domain-expert screening. Candidates retained after such screening may then be considered for subsequent experimental validation. This candidate pool also serves as a diagnostic record that can help identify overly restrictive variable bounds, incomplete constraints, insufficient population diversity, or other inappropriate optimization settings when satisfactory candidates are not obtained.

The pymoo-based mixed-variable evolutionary implementation is executed with a population size of 40–100 for 40–200 generations using the default mixed-variable mating and variation settings provided by pymoo.

## 3. Results and Discussion

### 3.1. Results

**Figure 2:**
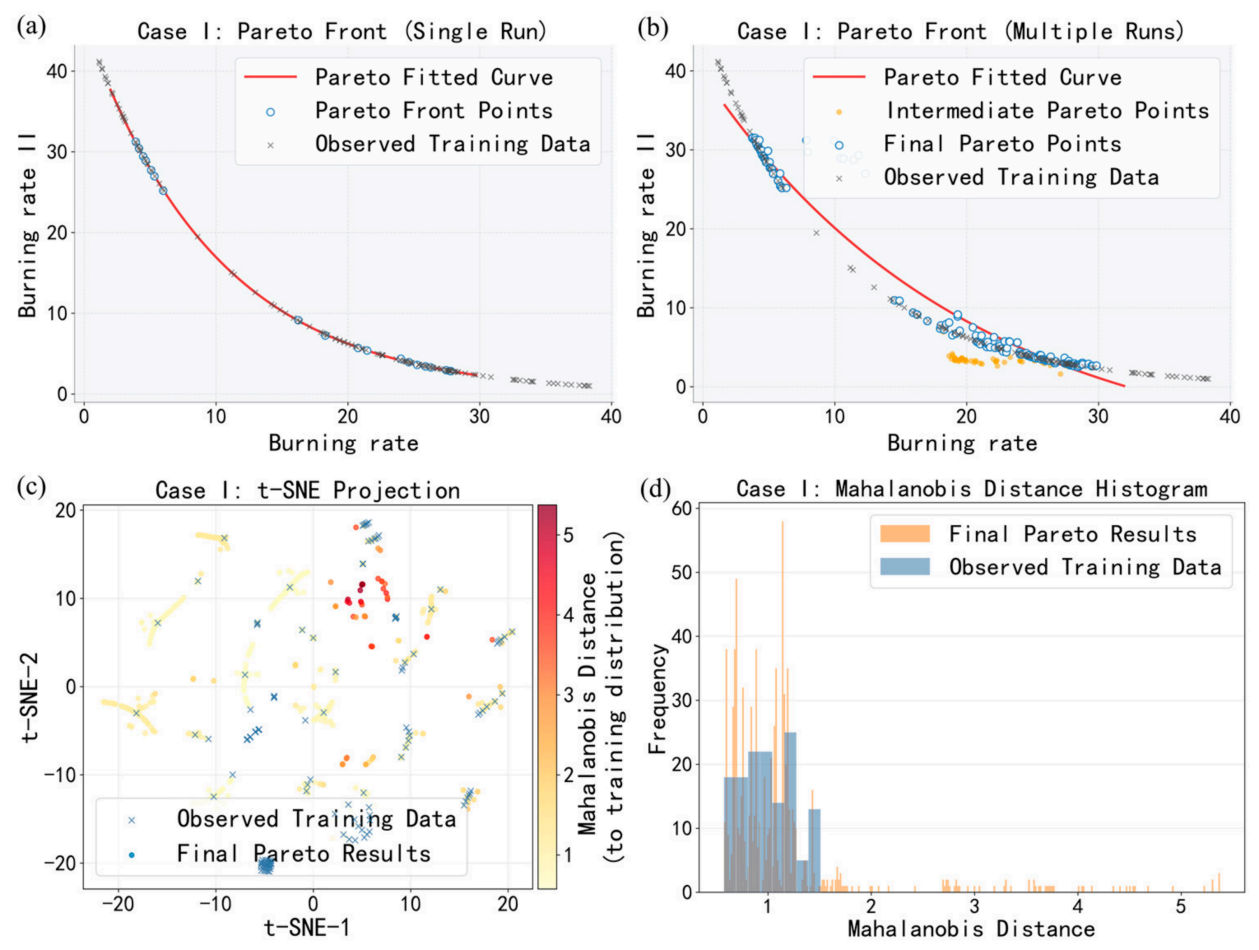
Optimization results for Case I obtained by NSGA. (**a**) Pareto front from a single run. Crosses (×) represent the observed data (historical formulations), circles (○) denote the Pareto solutions, and the red curve is the Pareto fitted curve. (**b**) Deduplicated final Pareto solutions and intermediate Pareto candidates collected across generations and multiple independent optimization runs. (**c**) t-SNE projection of the final Pareto solutions retained across multiple runs, colored by Mahalanobis distance to the observed data distribution (yellow: small distance, red: large distance). (**d**) Mahalanobis distance distributions of the observed data and final Pareto solutions.

NSGA is applied to simultaneously optimize Burning rate and Burning rate II in Case I. The single-run result for Case I is shown in Figure 2a. The two axes represent the two mathematically defined objectives. In the figure, crosses (×) indicate the observed data points (i.e., the historical formulations from Case I), whereas circles (○) mark the Pareto front solutions obtained by the algorithm. The red curve, referred to as the fitted Pareto curve, is fitted to the Pareto front points. Its trend closely aligns with the distribution of the observed data, suggesting that even a single run is able to capture the expected trade-off between the two targets.

In addition to the final Pareto front solutions, feasible intermediate Pareto candidates identified in their respective generations are also collected across multiple independent optimization runs. After duplicate candidates are removed, the resulting deduplicated final/intermediate Pareto points for Case I is shown in Figure 2b, which are marked as blue open circles (○) and yellow filled circles (●), respectively. This candidate archive contains a broader pool of feasible trade-off candidates encountered at different stages of the search. Many archived candidates remain close to the historical observations, whereas others deviate from the fitted trend curve and represent additional regions visited during optimization. Because some archived candidates may subsequently be dominated by solutions identified in later generations, the complete archive is interpreted as a candidate repository and a record of the search process rather than as the final Pareto front.

Figure 2c presents the t-SNE projection of the final Pareto results retained across multiple runs for Case I, excluding the intermediate Pareto candidates. The two axes represent the transformed t-SNE components. Points located close to each other generally exhibit more similar local feature patterns, whereas the two axes themselves do not represent specific physical variables. Crosses (×) indicate the observed data points, while circles (○) denote the Pareto points. The color of each circle encodes the Mahalanobis distance of that candidate from the distribution of the observed data, calculated in the original feature space, with yellow indicating smaller distances and red indicating larger distances. The t-SNE map shows that the observed samples and Pareto candidates are distributed relatively sparsely and form several small clusters. Most clusters containing observed samples are surrounded by Pareto candidates with relatively small Mahalanobis distances. These candidates are therefore more similar to the observed samples and represent relatively conservative search results. In contrast, some local clusters of observed samples contain few or no Pareto candidates, suggesting that these neighborhoods are less represented among the retained candidates. Several local clusters consist predominantly of Pareto candidates and contain few nearby observed samples. These candidates also exhibit relatively large Mahalanobis distances, indicating greater statistical deviation from the historical data distribution. They are therefore interpreted as statistically more distant candidates whose predictions require greater caution.

Figure 2d presents the Mahalanobis-distance distributions of the Pareto results (yellow) and the historical observations (blue). The horizontal axis represents the Mahalanobis distance, while the height of each bar indicates the frequency of samples within the corresponding distance interval. The Mahalanobis distances of the Pareto results extend to approximately 5.5, while those of the observed data are primarily concentrated below approximately 1.6. This observation is consistent with the pattern revealed in Figure 2c: a subset of the Pareto results remains similar to the historical formulations, whereas the rest shows greater statistical deviation from the historical data distribution.

**Figure 3:**
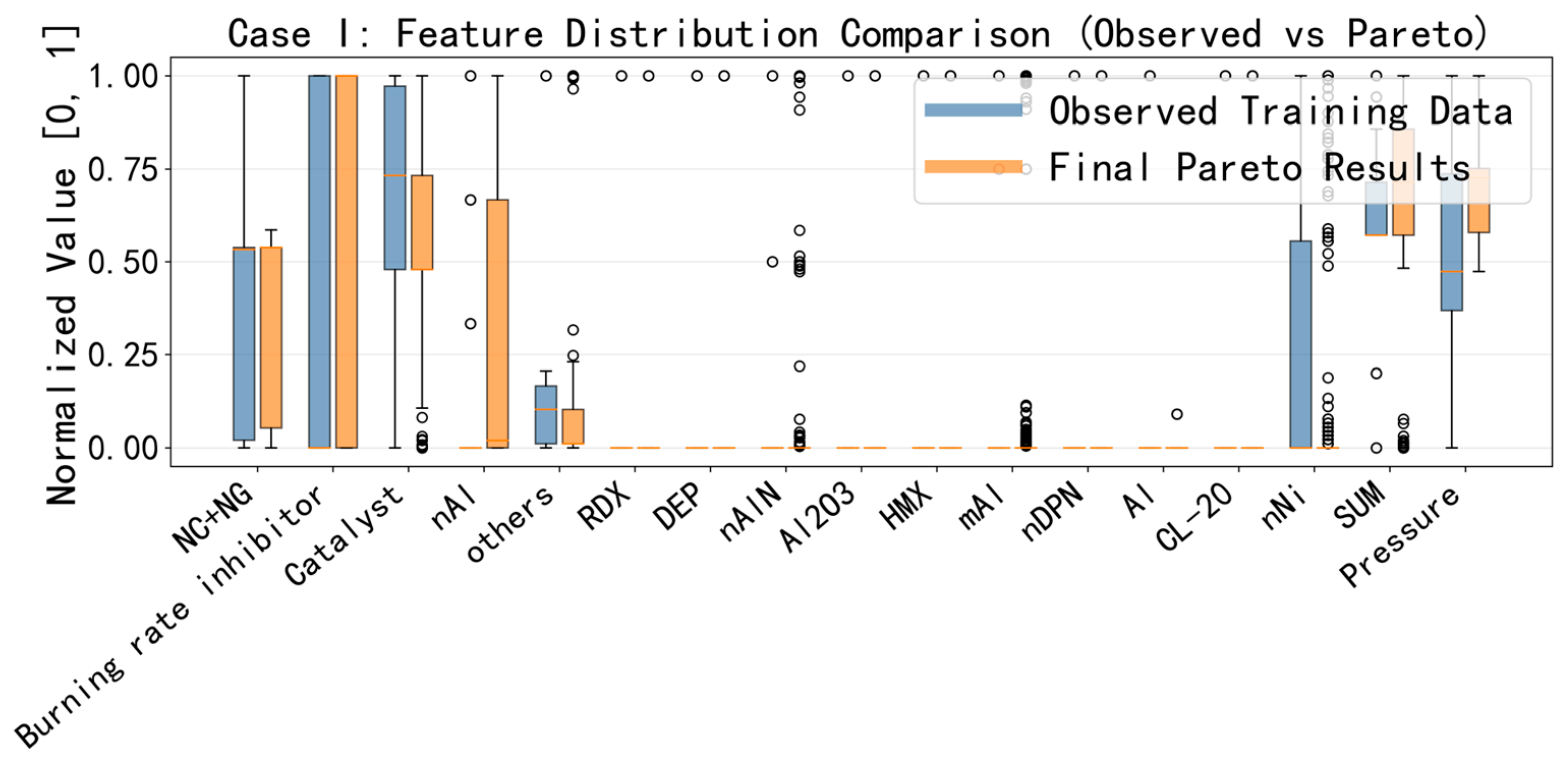
Feature distribution comparison between the observed data (blue) and the Pareto results (yellow) for Case I. The circles represent outliers beyond the whiskers of the boxplots.

Figure 3 compares the feature distributions of the historical observations (blue) and the Pareto results (yellow). In each box plot, the central line indicates the median, the box represents the interquartile range, the whiskers extend to values within 1.5 times the interquartile range, and the individual circles indicate values beyond the whiskers. For several features, including Burning rate inhibitor, RDX, and DEP, the distributions of the Pareto results are broadly consistent with those of the historical observations. Other features show either narrower or broader distribution ranges. For example, the central distribution range of NC + NG narrows from approximately 0–60% in the historical observations to 40–60% in the Pareto results. Similarly, the range of Catalyst narrows from approximately 50–100% to 50–80%, while that of Others narrows from 0–20% to 0–10%. In contrast, nAl, which is predominantly concentrated at 0 in the historical observations, expands to approximately 0–65% in the Pareto results. The distribution of nNi narrows from approximately 0–60% to values predominantly concentrated at 0. The range of SUM expands from approximately 55–70% to 55–85%, whereas the distribution of Pressure shifts toward higher values, from approximately 35–80% to 50–85%. Overall, the distributional comparison indicates that the optimization maintains substantial similarity to the historical formulation space for many features while selectively concentrating or extending the ranges of others. The narrowed or shifted distributions reflect the regions favored under the specified objectives and constraints, whereas the expanded distributions indicate exploration beyond the densely represented historical ranges. The observed variability is methodologically expected because continuous variables can be searched throughout their configured bounds, whereas discrete variables are restricted to predefined admissible values. The box plots are used descriptively to compare the candidate distributions selected by the objectives and constraints with the historical data and are not interpreted as estimates of random sampling uncertainty.

**Figure 4:**
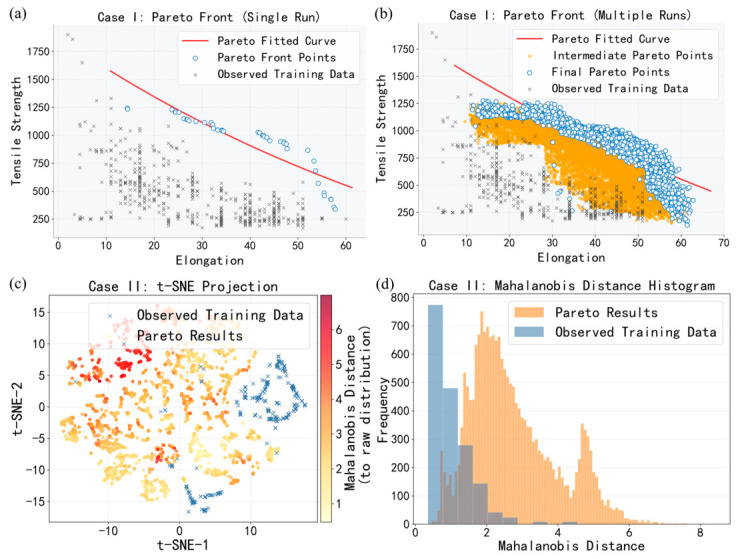
Optimization results for Case II obtained by NSGA. (**a**) Pareto front from a single run. Crosses (×) indicate observed data, circles (○) mark Pareto solutions, and the red curve is the fitted curve. (**b**) Deduplicated final Pareto solutions and intermediate Pareto candidates collected across generations and multiple independent optimization runs. (**c**) t-SNE projection of the final Pareto solutions retained across multiple runs, colored by Mahalanobis distance (yellow: small distance, red: large distance). (**d**) Mahalanobis distance distributions of the observed data and the final Pareto solutions.

Figure 4a presents the single-run result for the Case II. The historical data, marked by black crosses (×), are broadly distributed in the objective space defined by elongation and tensile strength, with a substantial proportion concentrated in regions of relatively low tensile strength. The Pareto front solutions, shown as blue open circles (○), are generally located along the upper boundary of the historical data. The fitted Pareto curve exhibits a downward trend, indicating that an increase in elongation is generally accompanied by a reduction in the maximum attainable tensile strength. This result captures the expected strength–ductility trade-off and demonstrates that a single optimization run identifies candidate alloy compositions representing different compromises between the two properties.

Figure 4b presents the deduplicated final and intermediate Pareto points collected across multiple independent optimization runs for Case II. The final Pareto points, shown as blue open circles (○), form a relatively continuous upper boundary across a broad range of elongation and tensile strength, demonstrating the trade-off between the two objectives. The intermediate Pareto points, shown as yellow filled circles (●), occupy a broader region below this boundary and record alternative candidates encountered during the optimization process. Compared with the single-run result, the multiple-run results provide denser coverage of the objective space and a broader candidate pool for domain-expert screening. As in Case I, the intermediate candidates are treated as a record of the search process rather than as part of the final Pareto front.

Figure 4c presents the t-SNE projection of the final Pareto results retained across multiple runs for Case II, excluding the intermediate Pareto points. The historical observations form several relatively compact clusters, particularly on the right side of the projection, whereas the Pareto candidates occupy a broader region and form multiple branches and clusters. Some Pareto candidates are located near the historical observations and exhibit relatively small Mahalanobis distances, indicating greater statistical similarity to the historical compositions. Because t-SNE preserves local rather than global structure, the apparent separation between candidates and historical observations is interpreted only qualitatively. Candidates with larger Mahalanobis distances show greater statistical deviation from the historical data distribution and therefore require more cautious interpretation and subsequent domain-expert assessment.

Figure 4d quantitatively supports the distributional pattern observed in the t-SNE projection. The Mahalanobis distances of the historical observations are primarily concentrated below approximately 2.5, whereas those of the Pareto results extend from approximately 0.5 to 8. The overlap between the two distributions indicates that some Pareto candidates remain similar to the historical alloy compositions. However, the broader distribution and longer upper tail of the Pareto results show that a substantial proportion of the candidates extend into less represented regions of the composition space. These results demonstrate that the optimization generates both relatively conservative candidates and more exploratory candidates. Nevertheless, a large Mahalanobis distance indicates statistical deviation rather than physical infeasibility, and these candidates require further evaluation based on metallurgical knowledge and experimental validation.

**Figure 5:**
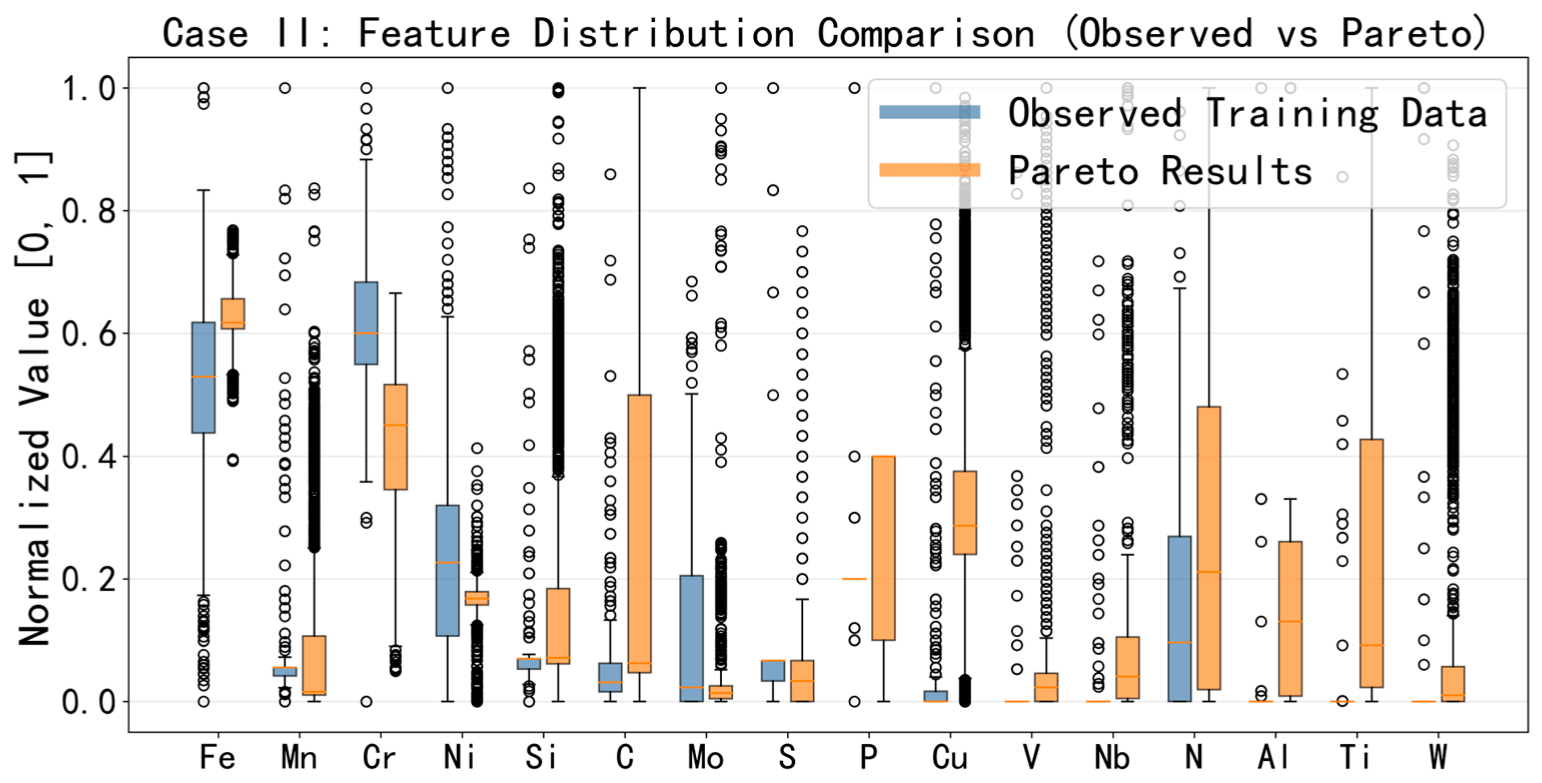
Feature distribution comparison between the observed data (blue) and the Pareto results (yellow) for Case II. The circles represent outliers beyond the whiskers of the boxplots.

Figure 5 compares the normalized feature distributions of the historical observations (blue) and the Pareto results (yellow) for Case II. Several features exhibit clear distributional changes after optimization. The distribution of Fe becomes narrower and shifts toward higher values, whereas that of Cr shifts toward lower values. Ni also occupies a narrower range in the Pareto results. In contrast, C exhibits a substantially broader distribution, while Mo becomes more concentrated near its lower range. The distributions of Cu, N, Al, and Ti also extend across broader ranges than those of the historical observations. In particular, Cu, Al, and Ti are concentrated predominantly near their lower bounds in the historical data but occupy wider ranges in the Pareto results. P is concentrated at a specific normalized level, reflecting the discrete-variable configuration used in the optimization. Other features, including Mn, Si, S, V, Nb, and W, remain primarily concentrated in relatively low-value regions, although their distribution ranges differ to varying degrees. These distributional changes indicate that the optimization does not simply reproduce the historical alloy compositions. Instead, it selectively favors narrower composition ranges for some elements while extending other elements into regions that are less densely represented in the historical data. The narrowed or shifted distributions reflect composition regions favored under the elongation and tensile-strength objectives and the specified constraints, whereas the expanded distributions indicate greater exploration of the alloy composition space. As in Case I, this variability reflects the configured mixed-variable search space together with objective- and constraint-driven selection.

### 3.2. Discussion

As discussed in the previous sections, recent ML-assisted chemical-design approaches use pre-trained large language models, large-scale datasets, or extensive candidate spaces to support materials exploration [28,29,30], including the design of new molecular structures and the optimization of experimental conditions. However, the direct application of similar approaches may be challenging when only small datasets are available and the design variables are subject to multiple constraints. For example, Cases I and II in the present study contain only 137 and 587 records, respectively, and involve both continuous and discrete variables as well as composition-related equality and inequality constraints. Under these conditions, an important task is to translate the available data and practical design requirements into an appropriate optimization problem. The present work therefore focuses on configuring mixed continuous–discrete variables, multiple predicted objectives, and domain constraints within an executable multi-objective optimization framework. Continuous variables are assigned numerical ranges, discrete variables are restricted to predefined admissible values, and equality and inequality constraints are represented by numerical residuals. NSGA is then used to search the configured formulation or composition space.

The two cases illustrate the application of this framework under different conditions. Case I provides a controlled demonstration of the workflow for a small energetic-material formulation dataset, while Case II considers the practical trade-off between independently measured elongation and tensile strength in a steel-alloy dataset. In both cases, the framework generates final Pareto solutions under the specified variable ranges and constraints. The retained intermediate Pareto candidates additionally provide a broader candidate pool and a record of the regions visited during optimization.

Overall, these results show that multi-objective optimization can be applied to small-sample formulation-design problems by appropriately configuring the available variables, predicted properties, and domain constraints. The statistical distribution analyses further help distinguish candidates close to the historical data from those requiring greater extrapolation by the predictive models. These exploratory candidates do not necessarily represent chemically or experimentally valid formulations and therefore require domain-expert assessment and subsequent experimental validation. Additional NN-distance, mean five-nearest-neighbor-distance, and bootstrap-uncertainty analyses for both cases are provided in Section S1 and Figures S1–S4 of the Supporting Information. Nevertheless, the framework provides a practical connection between data-driven prediction and constrained candidate screening in small-sample formulation design.

## 4. Conclusions and Outlooks

In this work, a problem-setup-centric optimization framework for mixed-variable formulation design is developed and systematically evaluated on an energetic-material formulation dataset and a steel-alloy composition dataset. Instead of introducing a new optimization algorithm, the study focuses on rigorously defining the optimization problem, including mixed continuous–discrete variable representation with customized continuous variable bounds derived from historical data and domain-informed admissible sets for discrete variables, constraint formulation, and objective construction, and integrating these elements into a multi-objective genetic algorithm based on pymoo.

Across both Case I and Case II, the proposed framework consistently generates feasible Pareto-optimal solutions while preserving all equality and inequality constraints. The obtained Pareto fronts closely follow the trends observed in historical data, confirming that the framework effectively captures underlying structure in the formulation–performance relationship. At the same time, some generated candidates show greater statistical deviation from the historical data distribution, providing a broader candidate pool for domain-expert assessment and subsequent experimental validation.

These findings indicate that a general-purpose multi-objective optimizer can support small-sample formulation design when mixed variable types, multiple predicted objectives, and domain constraints are explicitly configured. In practical applications, the final Pareto solutions provide the identified objective trade-offs, while the intermediate Pareto candidates provide a broader candidate pool for domain-expert screening and a record for diagnosing the optimization process.

Despite these promising results, several directions remain for future work. First, the current objective formulation relies on surrogate machine learning models and a deterministic error-based objective; future research could incorporate uncertainty-aware objectives or physics-informed constraints to improve robustness in real-world applications. Second, while the current pymoo-based evolutionary implementation provides a practical search engine for the configured formulation problems, more advanced or hybrid multi-objective optimizers (e.g., adaptive, surrogate-assisted, or reinforcement learning–guided evolutionary algorithms) may further enhance search efficiency, especially for higher-dimensional formulation spaces.

Third, the current discrete variable encoding is based on predefined categorical mappings; extending the framework to handle hierarchical or conditional discrete structures could broaden its applicability to more complex industrial formulation problems. In addition, integrating active learning strategies to iteratively update surrogate models using newly generated Pareto solutions would enable a closed-loop optimization–data acquisition pipeline, improving predictive accuracy over time.

Finally, although the present study identifies candidates with varying degrees of statistical deviation from the historical data distribution, systematic validation through experiments or high-fidelity simulations remains an important step toward practical deployment. Such validation would be essential for confirming the physical realizability and performance advantages of newly suggested formulations.

Overall, this study establishes a flexible and generalizable optimization workflow for mixed-variable formulation design and provides a foundation for future extensions toward more adaptive, data-efficient, and physics-consistent formulation optimization frameworks.

## Data Availability

The data presented in this study are openly available in Study on the Thermal Decomposition Characteristics and Combustion Performance of HTPB/AP/Al/RDX Propellants at https://d.wanfangdata.com.cn/thesis/ChJUaGVzaXNOZXdTMjAyMDEwMjgSB1kzNzE2OTgaCDFldmo1cW9q accessed on 20th May 2026.

## Supplementary Materials

The following supporting information can be downloaded at: https://www.mdpi.com/article/10.3390/ma19153248/s1, Figure S1: Distributions of the nearest-neighbor distance and mean five-nearest-neighbor distance for the final Pareto solutions and historical training data in Case I. The historical reference distances are calculated using a leave-one-out procedure. Figure S2: Nearest-neighbor distance versus bootstrap prediction standard deviation for the final Pareto solutions and historical training data in Case I. The dashed and dotted lines indicate the median and 95th percentile, respectively, of the uncertainty estimates for the historical training data. Figure S3: Distributions of the nearest-neighbor distance and mean five-nearest-neighbor distance for the final Pareto solutions and historical training data in Case II. The historical reference distances are calculated using a leave-one-out procedure. Figure S4: Nearest-neighbor distance versus bootstrap prediction standard deviation for the final Pareto solutions and historical training data in Case II. The dashed and dotted lines indicate the median and 95th percentile, respectively, of the uncertainty estimates for the historical training data.
