# Supplementary material for: A Problem-Setup-Centric Framework for Mixed-Variable Chemical Formulation Optimization with Customized Constraints
Source: Materials (Basel). 2026 Jul 31;19(15):3248. doi: 10.3390/ma19153248 (PMC13467574; doi:10.3390/ma19153248)
Supplement: Supplementary file 1 [file materials-19-03248-s001.zip › materials-4427325-supplementary.pdf]

S1. Training-Space Distance and Bootstrap Uncertainty Analyses

To complement the Mahalanobis-distance and t-SNE analyses, nearest-neighbor (NN) distance and mean k-nearest-neighbor (kNN) distance are calculated in the standardized feature space using scaling parameters derived from the historical training data. For each final Pareto solution, the NN distance represents its distance to the closest historical training sample, whereas the kNN mean distance represents its average distance to the five closest historical samples. The corresponding distances of the historical samples are calculated using a leave-one-out procedure. In addition, prediction uncertainty is estimated by training 40 XGBoost models on independently bootstrap-resampled training datasets and calculating the standard deviation of their predictions. The median and 95th percentile of the uncertainty estimates for the observed training data are included as reference levels.

S1.1. Case I

For Case I, the NN-distance distribution shows that a large proportion of the final Pareto solutions are located close to at least one historical training sample. This result indicates that many of the generated solutions retain feature combinations similar to existing formulations. Nevertheless, the distribution also exhibits a long upper tail extending to substantially larger distances, indicating that a smaller subset of the final Pareto solutions deviates considerably from its closest historical sample.

The kNN mean-distance distribution provides a more conservative assessment of the local training-data coverage. Compared with the NN-distance distribution, it exhibits greater overlap between the historical observations and the final Pareto solutions in the main distribution range. This suggests that many final Pareto solutions are located not only near an individual historical sample but also within or close to locally represented regions of the historical dataset. However, the extended upper tail of the final Pareto solutions indicates that some candidates remain distant from the broader local neighborhoods formed by the historical data. Case I therefore contains both relatively conservative solutions and a smaller number of statistically more distant solutions.

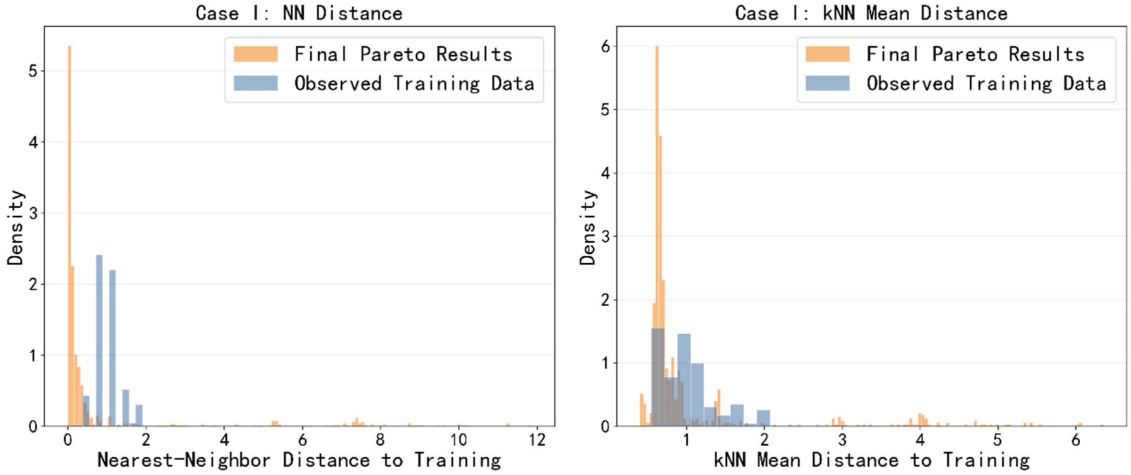

Figure S1. Distributions of the nearest-neighbor distance and mean five-nearest-neighbor distance for the final Pareto solutions and historical training data in Case I. The historical reference distances are calculated using a leave-one-out procedure.

The distance–uncertainty plot for Case I shows that most final Pareto solutions with small NN distances exhibit bootstrap uncertainty below or near the 95th-percentile reference value of the observed data (1.063). Several solutions at larger distances exhibit uncertainty above this reference level, indicating increased sensitivity of their predictions to bootstrap resampling. However, uncertainty does not increase monotonically with NN distance, and some statistically distant solutions retain relatively low bootstrap uncertainty. Training-space distance and bootstrap uncertainty therefore provide complementary information: the former characterizes statistical deviation from the historical feature distribution, whereas the latter characterizes the stability of the model predictions under data resampling.

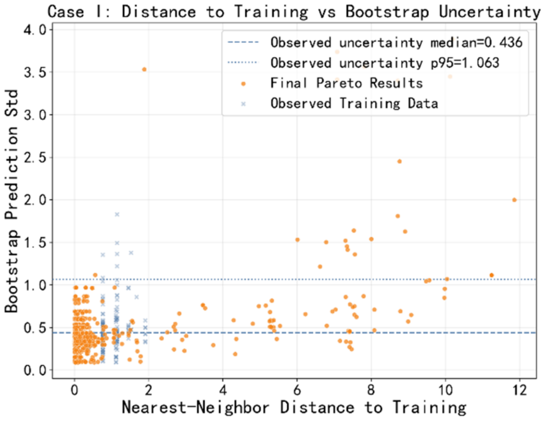

Figure S2. Nearest-neighbor distance versus bootstrap prediction standard deviation for the final Pareto solutions and historical training data in Case I. The dashed and dotted lines indicate

the median and 95th percentile, respectively, of the uncertainty estimates for the historical training data.

S1.2. Case II

For Case II, both the NN-distance and kNN mean-distance distributions of the final Pareto solutions shift markedly toward larger values relative to the historical training data. The NN distances of the historical observations are concentrated primarily in the lower range, whereas the final Pareto solutions are distributed mainly at substantially greater distances and exhibit a long upper tail. This result indicates that most optimized alloy compositions differ considerably from their closest historical compositions.

A similar pattern is observed for the kNN mean distance. The historical samples are concentrated predominantly at relatively small distances, whereas the final Pareto solutions occupy a broader and right-shifted distribution. The limited overlap between the two distributions indicates that a subset of the final Pareto solutions remains relatively close to historically represented composition regions. However, the majority exhibit greater statistical deviation from the local neighborhoods of the historical alloy dataset. Compared with Case I, Case II therefore produces a larger proportion of solutions located outside the densely represented regions of the training space.

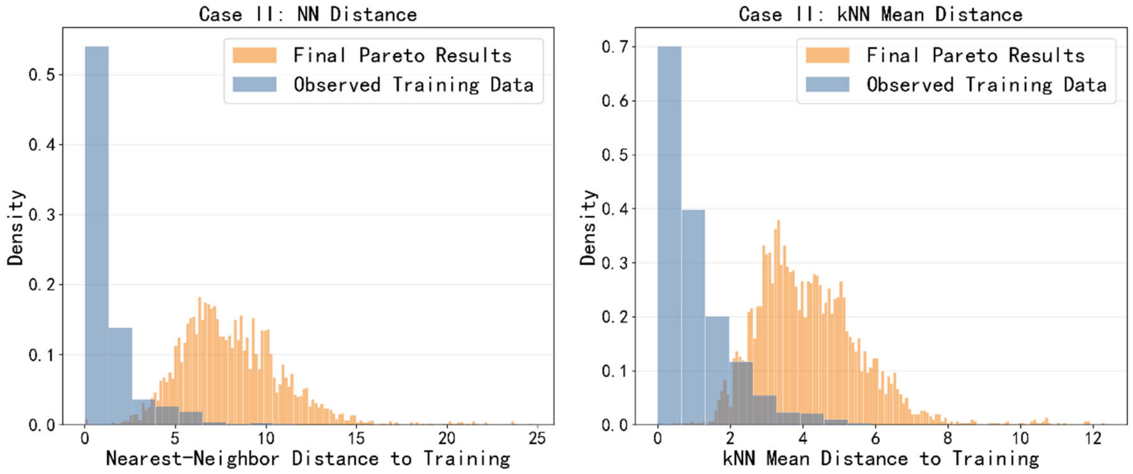

Figure S3. Distributions of the nearest-neighbor distance and mean five-nearest-neighbor distance for the final Pareto solutions and historical training data in Case II. The historical reference distances are calculated using a leave-one-out procedure.

The Case II distance–uncertainty plot further shows that many final Pareto solutions exhibit bootstrap uncertainty above the observed-data median of 27.577, and a considerable number also exceed the observed-data 95th-percentile reference value of 84.623. This result indicates that the

predictions for many optimized alloy compositions are more sensitive to bootstrap resampling than predictions for the majority of the historical samples. Nevertheless, the most distant solutions do not consistently exhibit the highest uncertainty, and no simple monotonic relationship is observed between NN distance and bootstrap prediction standard deviation. The two indicators should therefore be interpreted jointly rather than interchangeably.

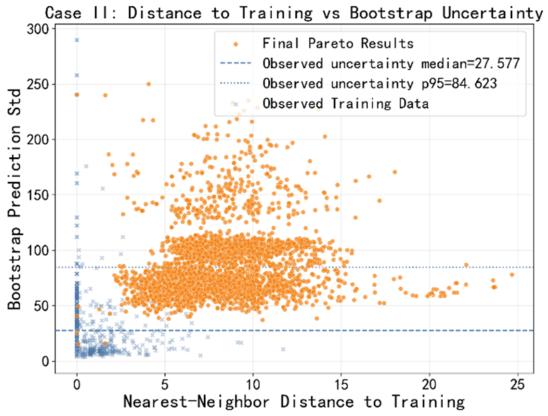

Figure S4. Nearest-neighbor distance versus bootstrap prediction standard deviation for the final Pareto solutions and historical training data in Case II. The dashed and dotted lines indicate the median and 95th percentile, respectively, of the uncertainty estimates for the historical training data.

Overall, the NN and kNN analyses provide additional quantitative measures of the distance between the final Pareto solutions and the historical training space, while the bootstrap analysis provides a complementary estimate of prediction stability. These results support the distinction between relatively conservative solutions and statistically more distant solutions but do not establish the chemical or engineering validity of the generated candidates. Candidates exhibiting large training-space distances, high bootstrap uncertainty, or both require more cautious interpretation, domain-expert assessment, and subsequent experimental validation.
